# *Achillea moschata* Wulfen: From Ethnobotany to Phytochemistry, Morphology, and Biological Activity

**DOI:** 10.3390/molecules27238318

**Published:** 2022-11-29

**Authors:** Martina Bottoni, Giovanna Baron, Francesca Gado, Fabrizia Milani, Laura Santagostini, Lorenzo Colombo, Paola Sira Colombo, Elisabetta Caporali, Alberto Spada, Marco Biagi, Claudia Giuliani, Piero Bruschi, Giancarlo Aldini, Gelsomina Fico

**Affiliations:** 1 Department of Pharmaceutical Science, University of Milan, 20133 Milan, Italy; 2 Ghirardi Botanical Garden, Department of Pharmaceutical Science, University of Milan, 25088 Toscolano Maderno, Italy; 3 Department of Chemistry, University of Milan, 20133 Milan, Italy; 4 Department of Biosciences, University of Milan, 20133 Milan, Italy; 5 Department of Agricultural and Environmental Sciences—Production, Landscape, Agroenergy, University of Milan, 20133 Milan, Italy; 6 Department of Physical Sciences, Hearth and Environment, University of Siena, 53100 Siena, Italy; 7 Department of Agricultural, Environmental, Food and Forestry Science and Technology, University of Florence, 50121 Florence, Italy

**Keywords:** primary data, traditional decoction, flower heads, aqueous and methanolic extracts, mass spectrometry, antioxidant activity, anti-inflammatory activity, glandular *indumentum*, microscopy

## Abstract

A multidisciplinary investigation on *Achillea moschata* Wulfen (Asteraceae) is outlined herein. This work, part of the European Interreg Italy–Switzerland *B-ICE* project, originated from an ethnobotanical survey performed in Chiesa in Valmalenco (Sondrio, Lombardy, Northern Italy) in 2019–2021 which highlighted this species’ relevance of use in folk medicine to treat gastrointestinal diseases. In addition, this contribution included analyses of the: (a) phytochemical profile of the aqueous and methanolic extracts of the dried flower heads using LC-MS/MS; (b) morpho-anatomy and histochemistry of the vegetative and reproductive organs through Light, Fluorescence, and Scanning Electron Microscopy; (c) biological activity of the aqueous extract concerning the antioxidant and anti-inflammatory potential through cell-based in vitro models. A total of 31 compounds (5 phenolic acids, 13 flavonols, and 13 flavones) were detected, 28 of which included in both extracts. Covering and secreting trichomes were observed: the biseriate 10-celled glandular trichomes prevailing on the inflorescences represented the main sites of synthesis of the polyphenols and flavonoids detected in the extracts, along with volatile terpenoids. Finally, significant antioxidant and anti-inflammatory activities of the aqueous extract were documented, even at very low concentrations; for the first time, the in vitro tests allowed us to formulate hypotheses about the mechanism of action. This work brings an element of novelty due to the faithful reproduction of the traditional aqueous preparation and the combination of phytochemical and micromorphological research approaches.

## 1. Introduction

*Achillea moschata* Wulfen (Asteraceae) is a suffruticose chamaephyte. It is endemic to the Alpine region and grows between 1400 m and over 3000 m a.s.l. on cliffs, stony ground, and moraines, exclusively on silica. It is known by various common and vernacular names, including *erba iva*, *taneda*, *daneda*, and *aneda*. The decoction of its flower heads, which represents the drug, is traditionally used to treat digestive tract disorders [1]. The balsamic time coincides with anthesis (July–August) [1].

**Figure 1:**
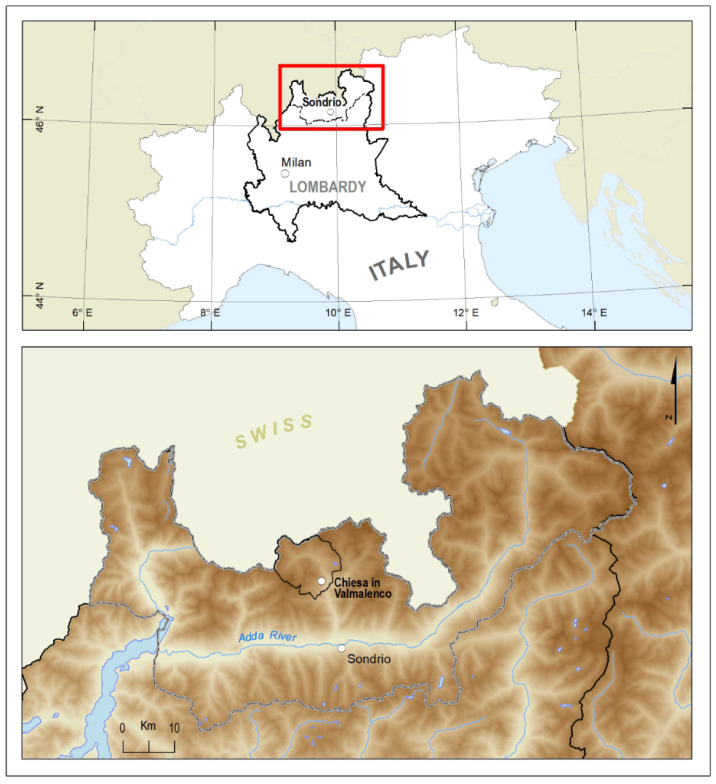
Geographical position of Chiesa in Valmalenco (Sondrio, Lombardy, Northern Italy). Original map by L. Dell’Olmo, University of Florence.

This study is derived from an ethnobotanical survey related to the European Interreg Italy–Switzerland *B-ICE* project. The main purpose of this project is to create a management model useful in dealing with emerging issues in Valmalenco (Sondrio, Lombardy, Northern Italy) due to climate change. Glacial retreat and diminished snowfall are adversely affecting tourism in the area. Therefore, the *B-ICE* management model includes strategies aimed at looking for new tourism avenues, such as the revitalization of traditional practices linked to medicinal and food wild plants [1]. Specifically, our work focuses on Chiesa in Valmalenco (Figure 1), which is the main village of the Valley. It has about 2400 inhabitants, is located at approximately 1000 m a.s.l., and is enclosed by the Bernina Alps.

Previous ethnobotanical works that mentioned *A. moschata* referred to the Alpine and Pre-alpine regions, with special focus on the Italian territory [1,2,3,4,5,6]. Common aspects regarding the traditions of use emerged: from the employed plant parts (flower heads, flowered aerial parts, and flowered epigeal organs) to the most popular preparations (decoctions/infusions and digestive liqueurs). Folk uses in the Alpine and Pre-alpine regions mainly referred to a digestive, antispasmodic, and carminative action. They also mentioned antiseptic, emollient, anti-inflammatory, and painkiller properties, as well as hypotensive characteristics [1,2,3,4,5,6].

Concerning previous phytochemical studies, the literature proposed various works on the essential oil composition [7,8,9] and the polar extracts [9,10,11,12], as well as the non-polar and aqueous profiles [9,13], obtained from the dried flowered aerial parts. The acetonic extracts were also analyzed for the identification of the epicuticular components [14]. Finally, Apel et al. (2021) evaluated and compared the hydroalcoholic extracts (methanol/water) of three congeneric species: *A. millefolium*, *A. moschata*, and *A. atrata* [15].

The characterization of the aqueous extract proposed in the literature deserves further investigation, with morphological studies on the target species aimed at investigating the secreting structures responsible for productivity in secondary metabolites lacking. Indeed, previous micromorphological contributions focused on European and Western Asian congeneric species [16,17,18,19,20,21]. Regarding the biological activity, *A. moschata* extracts were generally screened for their radical scavenger properties with spectrophotometric assays such as the DPPH test [11,12]. Moreover, studies never reported the aqueous extract, which is significant, being the traditional decoction used in folk medicine in the Alpine and Pre-alpine regions. Only recently, Vitalini et al. [9] tested several extracts, including the aqueous one, for their anti-inflammatory activity, evaluating the modulation of interleukins 8 and 1β on CaCo-2 cells. Concerning congeneric species, the antioxidant potential of different extracts was evaluated as radical scavenger activity, but no in vitro studies were reported to date in the literature [10,22,23,24].

We started our work with a (1) pilot field ethnobotanical research. Its primary focus was the collection and preservation of primary data obtained directly from actual users, concerning *A. moschata*-based remedies and traditional practices. We then characterized the species by combining complementary research approaches. Specifically, we (2) examined the phytochemical profile of the aqueous and methanolic extracts of the dried flower heads, obtained according to the referred traditional practices, from samples at anthesis gathered at the study area; (3) described the micromorphology of the vegetative and reproductive organs, with a special focus on the drug and secretory structures responsible for the production of secondary metabolites; and (4) evaluated the antioxidant and anti-inflammatory activities of the aqueous extract using two different cell models.

## 2. Results and Discussion

### 2.1. Ethnobotanical Research

A total of 125 informants were interviewed, aged between 18 and 96 years old. They provided information on 148 plant species (Table S1), 74 of which were used therapeutically in humans (Table S2). Among these 74, the most cited were *Arnica montana* L. (16.7% of the total citations), *A. moschata* (8.9%), *Malva sylvestris* L. (7.8), *Pinus mugo* Turra (6.5%), and *Hypericum perforatum* L. (6.3%). Their use was deeply rooted in the malenca daily life. All ailments retrieved from the informants were tabulated in 18 categories of pathology [1]. Among these, musculoskeletal system disorders and traumas (27.0% of the total citations), respiratory tract infections (18.6%), digestive tract disorders (16.1%), and skin disease and traumas (8.4%) were the most cited. Focusing on the treatment of digestive tract discomforts, 28 species were employed and most of the malenca traditional knowledge in this field of application was concentrated on the use of a single plant species: *A. moschata*. In fact, of the total 239 citations for this category, 112 referred to this autochthonous plant, immediately followed by *Matricaria chamomilla* L. (*n* = 19), *Gentiana lutea* L. (*n* = 18), and *Artemisia genipi* Weber ex Stechm. (*n* = 13), which clearly represented a steep decline. For the treatment of these disorders, all of the informants were consistent in using the flower heads (rarely described as aerial parts) in the form of decoction to aid digestion, as a gastric antispasmodic, for abdominal pain, and stomachache. Within the eight ethnobotanical works conducted in the Alpine and Pre-alpine regions in the past 15 years [1,2,4,5,25,26,27,28], seven documented the traditional use of *A. moschata*. Further confirmation on the same traditional use of the species was obtained by five of those seven works [1,2,4,5,27]. Furthermore, they described the use of decoctions/infusions of the flowered epigeal organs as a digestive, antispasmodic, and carminative agents. On the other hand, Cornara et al. [26] documented the use of compresses for eye inflammation, while Bruschi et al. [28] simply reported the category DSD (Digestive System Disorders), without mentioning specific pathologies.

### 2.2. Phytochemical Investigation

**Figure 2:**
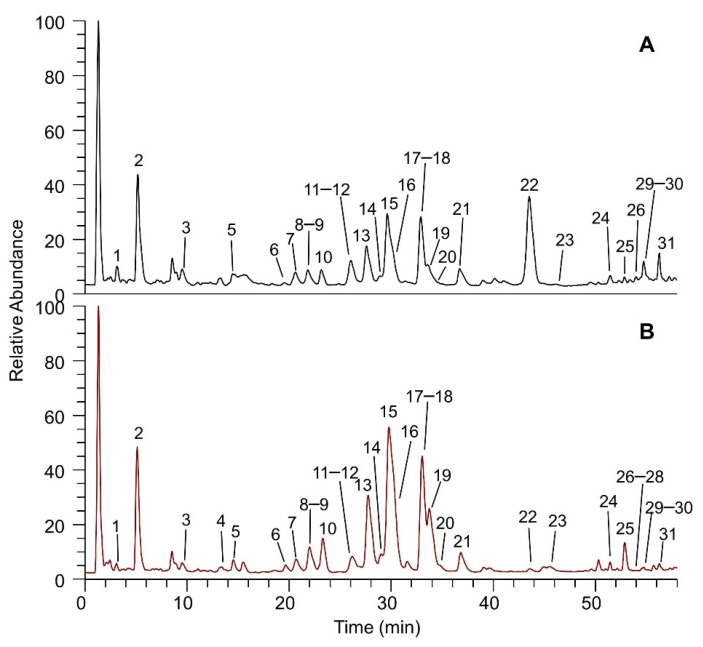
TICs of (**A**) aqueous extract and (**B**) methanolic extract of *Achillea moschata.* The peaks are numbered in progressive order based on the Retention Times (RT) and their assignment is reported in Table 1.

Regarding the phytochemical investigation, the qualitative profile of the aqueous and methanolic extract of the dried flowered aerial parts of *A. moschata* was evaluated. One of the elements of novelty offered by this work was the faithful reproduction of the traditional aqueous preparation, which was directly documented in the form of decoction through the ethnobotanical survey conducted in the study area. Another original aspect of this study concerned the characterization of the polyphenolic content of the traditional preparation, with the aim to better understand its potential in terms of bioactive compounds. Furthermore, this analysis was enriched with the characterization of the methanolic extract obtained by extraction with solvents of increasing polarity. This, in turn, facilitated a comparison with the literature data currently available about the target species, which mainly refer to this type of extract [10,11,12,15]. Figure 2 shows the Total Ion Currents (TICs) of the two extracts analyzed in negative ion mode, showing that all the main components of the mixture were identified.

Table 1 reports the name of the components characterized by their accurate mass, isotopic and fragmentation pattern (where available), both in negative and positive ion mode, and listed based on their elution order.

The LC-MS/MS analyses allowed for the identification of all the main compounds, detecting a qualitative overlap between aqueous and methanolic extracts. A total of 31 compounds were identified, 28 of which were detected in both extracts. To this end, luteolin C-glucoside (4), desmethoxycentaureidin (27), and isorhamnetin (28) were found only in the methanolic extract, even if only in trace amounts. Among the 31 total compounds (31 in negative ion mode, 27 in positive ion mode), 5 were phenolic acids, 13 were flavonols, and 13 were flavones. In detail, the phenolic acids were represented by caffeoylquinic acids or dicaffeoylquinic acids isomers (1, 2, 11, 13, 19), with all present in both extracts. Flavonols were detected as glucosides and aglycones, and they were quercetin (6, 29), kaempferol (7, 30), isorhamnetin (12, 14, 16, 28), mearnsetin (9, 10), syringetin (17), eupatolin (21), and chrysosplenol-D (or jaceidin) (31) derivatives. These were also detected in both extracts, except for isorhamnetin aglycone (28), which was found only in the methanolic extract. Analogously, flavones were detected as glucosides and aglycones of apigenin (3, 15, 25), apparently the most abundant component, luteolin (4, 8, 23), isoorientin (18), axillarin (24), chrysoeriol (or hispidulin) (26), and desmethoxycentaureidin (27). Finally, malonyl derivatives of luteolin (luteolin 7-malonyl-glucoside, 20) and apigenin (apigenin 7-malonyl-glucoside, 22) were identified in the two extracts. Special attention was paid to apigenin 7-malonyl-glucoside (22), whose peak has a higher relative abundance in respect to the other as opposed to the methanolic one, thus revealing an important feature of the traditional preparation, considering that the decoction extraction process adopted for the analysis was derived from the folk medicine.

Regarding the phytochemical profiling of the phenolic content of *A. moschata*, the literature proposed only five works. Two of these highlighted the quali-quantitative characterization of the methanolic extract [11,12]; one proposed the quali-quantitative analysis of the acetone/water extract, later solubilized in methanol/water [15]; one focused on the quantification of the total polyphenolic content of the methanolic fraction [10]; and, finally, a more recent work, proposed a quantitative comparison between the methanolic extract and the aqueous preparation, without presenting any chromatogram and fragmentation pattern of the listed compounds [9].

From a comparison with these papers [9,11,12,15], our phytochemical characterization identified the highest total number of compounds. The phenolic classes were found to be the same, including simple phenols (phenolic acids) and flavonoids (flavones and flavonols). Moreover, our identification exhibited 14 exclusive compounds, not detected in the other profiles: vicenin-2 (3), schaftoside (5), eupatolin (21), axillarin (24), chrysoeriol/hispidulin (26), isorhamnetin (28), quercetin-3,3′-dimethylether (29), 6-hydroxykaempferol-3,6-dimethylether (30), and chrysosplenol/jaceidin (31), as well as glycosides of syringetin (17), luteolin (4), and isoorientin 7- methyl ether (18). In addition, the detection of two malonic acid derivatives, luteolin 7-malonyl-glucoside (20) and apigenin 7-malonyl-glucoside (22), highlighted another unique feature of our profiles, both in the methanolic extract and the decoction. Specifically, the latter compound seems to be highly concentrated in the aqueous extract and, to the best of our knowledge, both molecules were previously identified only in congeneric species such as *A. millefolium, A. alpestris*, and *A. ceretanum* [29].

These phytochemical data deepened the field work conducted by our research group on the traditional uses of *A. moschata* in the very distribution area of the species. This aspect further distinguishes our survey from previous works, which based their phytochemical investigation on ethnobotanical data obtained in the scientific literature [9,11,12,15], or sometimes analyzing commercial forms of the herbal drug, instead of spontaneous ones [15].

### 2.3. Morphological Investigation

#### 2.3.1. Botanical Description

**Figure 3:**
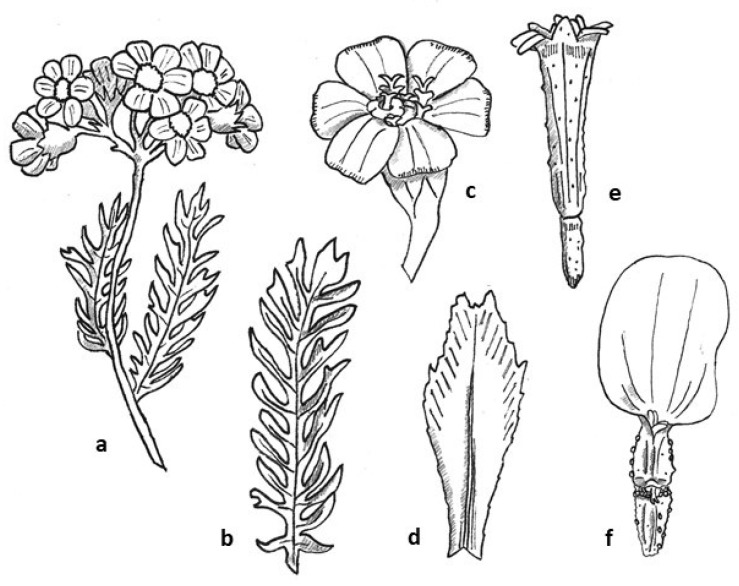
Botanical line drawings of *Achillea moschata* Wulfen: (**a**) Plant *in toto*. (**b**) Bipinnate leaf. (**c**) Flower head. (**d**) Involucral bract. (**e**) Disk floret. (**f**) Ray floret. Original drawings by L. Colombo.

*Achillea moschata* presents creeping woody stems from which ascending sterile shoots and erect flowering branches start (Figure 3). The latter are sub-glabrous or with short hairs. The leaves are alternate, deeply pinnate with a lanceolate–spatulate outline and 7–10 segments on each side (Figure 3). The basal leaves are pedicellate, while the cauline ones are sessile, toothed, and progressively linear. The leaves are strongly aromatic, hairless, or with scattered hairs [30]. The flowers are grouped in small flower heads (*capitula*) with a diameter of 2–6 mm, in turn gathered in loose corymbs, with each composed of about 10 flower heads (Figure 3). The peduncles of the flower heads are cylindrical, striated, sub-glabrous, or with scattered hairs [31]. The *capitula* are composed of both ray (6–9) and disk (10–15) florets. The receptacle is conic or convex, enclosed by various layers of imbricate involucral bracts. The bracts are obovate-oblong, sub-glabrous [31], with narrow transparent membranous margin, yellowish-to-brownish, and whole or notched only towards the apex with shallow denticles. The disk florets vary in number, from 10 up to 15 per head and are hermaphrodite. Each floret is subtended by an interfloral scale called “palea”. The corolla tube is yellow with the top split in 5 apical laciniae overhanging the tube. The stamens are 5 with free filaments and connate anthers, welded in a sleeve surrounding the stylus with deeply bifid stigma; the carpels are 2 forming a unilocular inferior ovary. The ray florets are 6–9 per head, pistillate, fertile, 3–4 mm long. The corollas are white, with a tubular lower portion surmounted by a ribbon-like extension, the ligule, ending with sub-round denticles, with scattered golden glands on the lower side [30]. The stigma is 2-lobed, with a papillose surface.

#### 2.3.2. Indumentum

**Figure 4:**
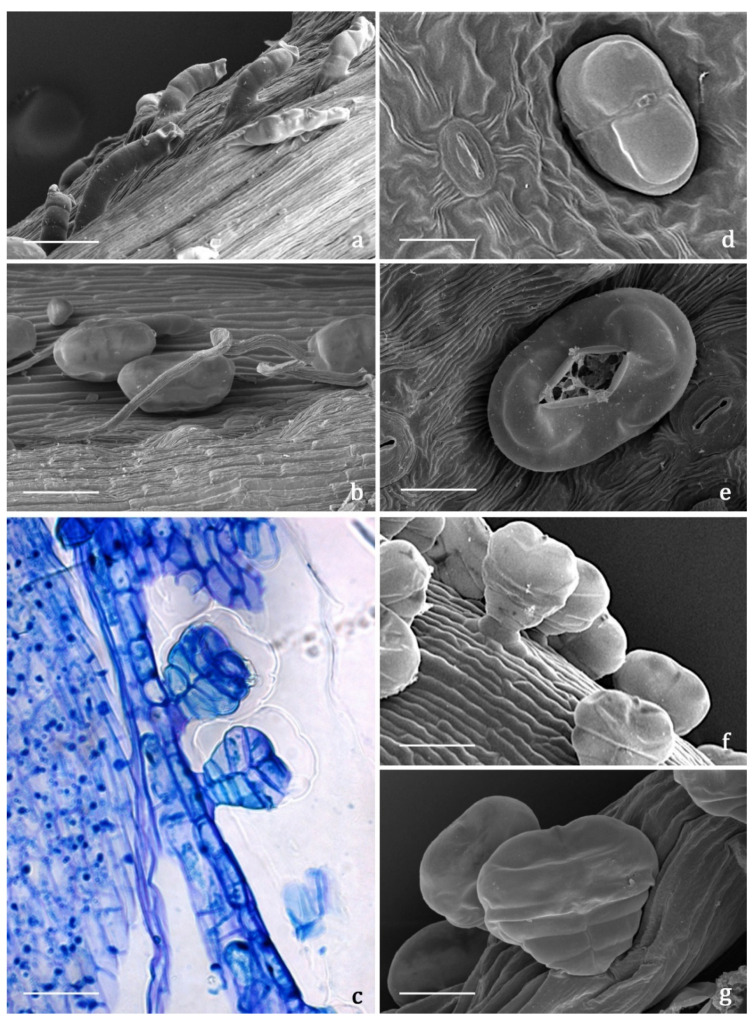
SEM and LM micrographs showing the covering and glandular trichomes observed on the vegetative and reproductive organs of *Achillea moschata*. (**a**) Short digitiform trichomes. (**b**) Long filamentous trichome and biseriate glandular trichomes. (**c**) Longitudinal section of biseriate 10-celled glandular trichomes stained with Toluidine Blue. (**d**) Sunken biseriate glandular trichome with intact cuticle. (**e**) Sunken glandular trichome showing the rupture of the cuticle along a line of weakness. (**f**,**g**) Protruding biseriate 10-celled glandular trichomes. Scale bars: 50 μm (**a–c**,**f**); 20 μm (**d**,**e**,**g**).

**Figure 5:**
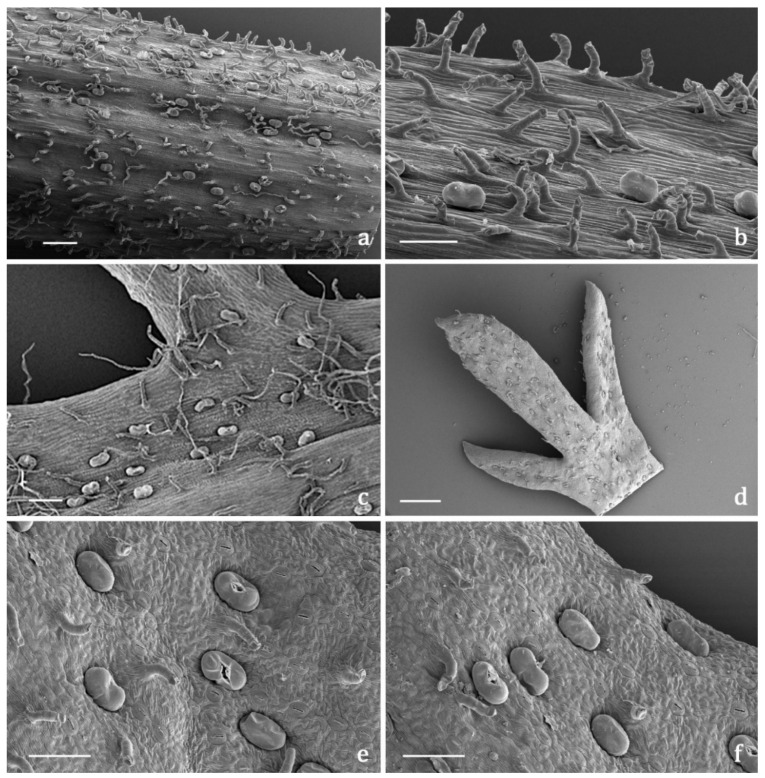
SEM micrographs showing the flowering stem and leaf morphology of *Achillea moschata*. (**a**,**b**) General view (**a**) and detail (**b**) of a flowering stem with biseriate glandular trichomes and digitiform covering trichomes. (**c**) Leaf abaxial surface with the two types of covering trichomes (short digitiform and long filamentous) and the biseriate glandular hairs. (**d**) General view of the apical portion of a deeply pinnate leaf (adaxial side). (**e**,**f**) Details of the leaf adaxial side with sunken biseriate glandular trichomes and digitiform hairs; notice the broken cuticle of several glandular trichomes for the release of the secreted materials. Scale bars: 500 μm (**a**,**d**); 100 μm (**b**,**c**,**e**,**f**).

**Figure 6:**
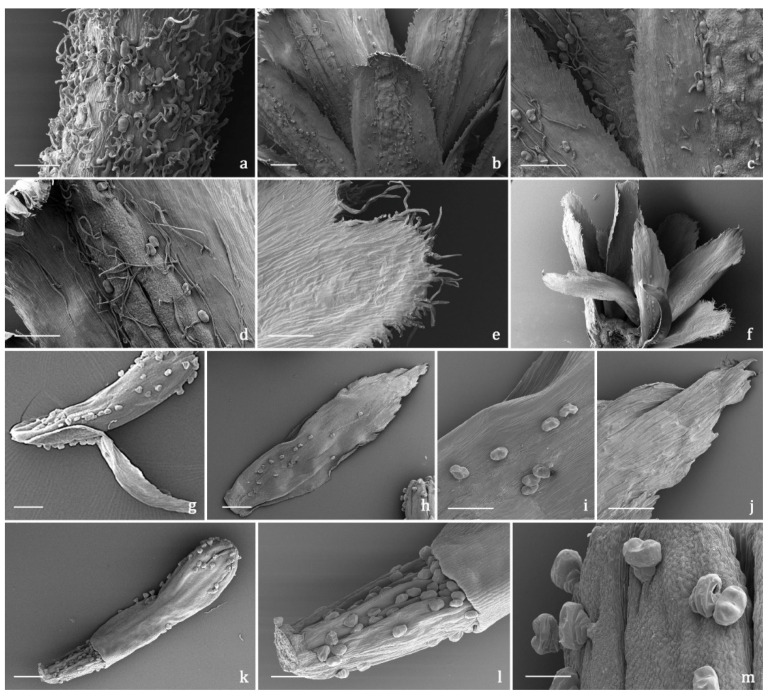
SEM micrographs showing the peduncle of the *capitulum* and the involucral bracts (**a**–**f**) and the disk florets (**g**–**m**) of *Achillea moschata*. (**a**) Peduncle of the *capitulum*. (**b**) General view of the involucral bracts (abaxial sides). (**c**,**d**) Details of the abaxial side of the involucral bracts with biseriate glandular trichomes and long filamentous hairs. (**e**) General view of the involucral bracts (adaxial sides). (**f**) Details of the apical portion of the abaxial side of an involucral bract. (**g**) General view of a disk floret subtended by an interfloral scale. (**h**–**j**) Interfloral scale: general view (**h**), details of the median portion (**i**), and of the apical portion (**j**) of the abaxial side. (**k**–**m**) Disk floret: general view (**k**), details of the ovary (**l**), and of the apical portion (**m**) of the corolla. Scale bars: 200 μm (**a**–**f**,**g**,**h**,**k**); 100 μm (**i**,**j**,**l**); 50 μm (**m**).

**Figure 7:**
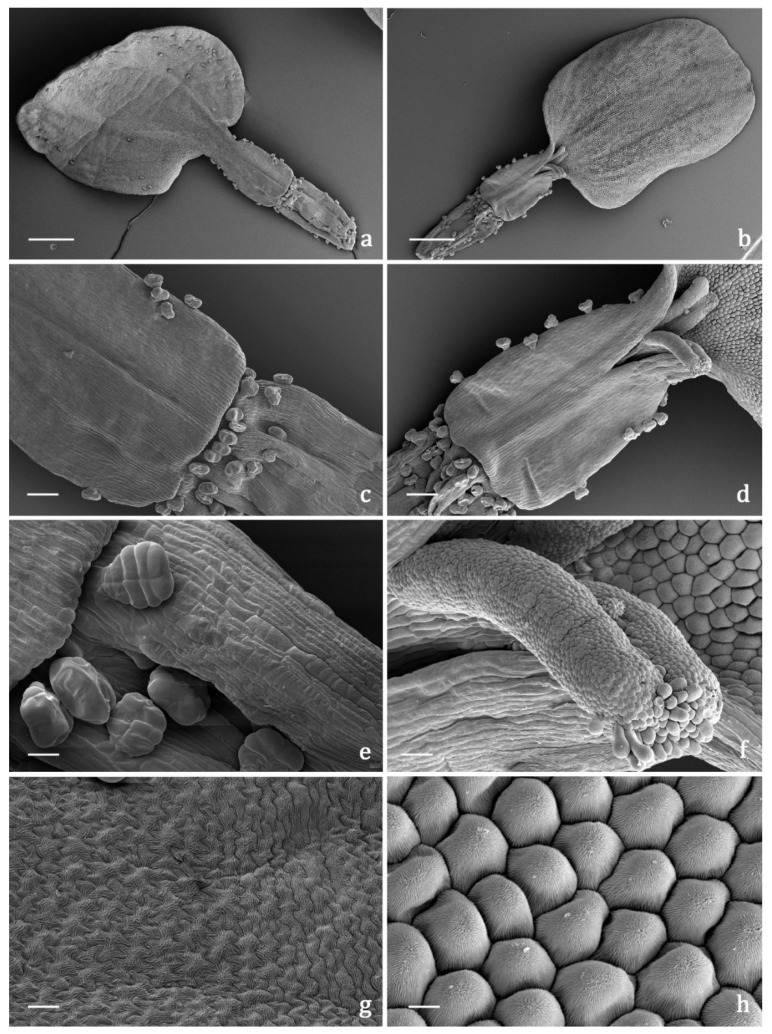
SEM micrographs showing the pistillate ray florets of the *capitulum* of *Achillea moschata*. (**a**,**b**) General view of a ray floret: abaxial (**a**) and adaxial (**b**) sides. (**c**,**d**) Details of the proximal portion of the corolla tube and of the distal portion of the ovary: abaxial (**c**) and adaxial (**d**) sides. (**e**) Biseriate, 10-celled glandular trichomes on the distal portion of the ovary. (**f**) Details of the 2-lobed stigma with a papillose surface. (**g**) Epidermis of the ligule adaxial side with elongated puzzle-like striated cells. (**h**) Epidermis of the ligule adaxial side with isodiametric conical striated cells. Scale bars: 500 μm (**a**,**b**); 100 μm (**c**,**d**); 20 μm (**e**,**f**); 10 μm (**g**,**h**).

The *indumentum* of the examined organs in all the replicates showed a general consistency both in terms of trichome morphotypes and distribution pattern (Figure 4, Figure 5, Figure 6 and Figure 7). Both covering and glandular hairs were observed (Figure 4). The formers were simple, multicellular, uniseriate, and distinguished in two subtypes: short and 5–7 celled with isodiametric cells of uniform diameter from the base to the apex, so as to appear digitiform (Figure 4a); long and filamentous with a pointed apex which may be straight or slightly twisted (Figure 4b). Although the cell number could not be defined based on LM and SEM analyses, the literature data indicated the occurrence of 5-celled filamentous hairs in some congeneric species [15,17]. The basal cells of both subtypes were usually protruding from the epidermal surface of the organs bearing them, and sporadically appeared swollen. The short digitiform hairs were distributed on flowering stems and on both leaf sides (Figure 5a–f), while the long filamentous ones were on the leaf abaxial sides, on the abaxial side of the involucral bracts, and on peduncles of the *capitulum* (Figure 5c and Figure 6a–d). The glandular trichomes belonged to a single morphotype (Figure 4b–g) that was a 10-celled biseriate, composed of two rows of five cells each (Figure 4c,f,g). There are two basal cells, two stalk cells, and six secretory cells, as already described in some congeneric species [19,20]. At full growth, the mature glands presented a peculiar bilobed, heart-like shape, resulting from the expansion of the cuticle covering the apical cells. In fact, during development, the subcuticular space at the apex grew into a sac-like structure, although it was unclear whether the sac embraced four or six apical cells (Figure 4f,g). These hairs may be sunken or may protrude to varying degrees from the epidermal surface because of the different elongation of the basal cells (Figure 4d–g). They appeared sunken on the flowering stems, leaves, involucral bracts, and interfloral scale of the disk florets (Figure 5a–f and Figure 6a–d,g–j), but protruded from the epidermis of the corolla of both disk and ray florets and on their ovaries (Figure 6k–m and Figure 7a–f). On the abaxial surfaces of involucral bracts, trichomes presented a peculiar distribution pattern: filamentous glands and trichomes were arranged in two rows in troughs along either side of the leaf midrib (Figure 6b–d). Most of the secretion was discharged externally following the rupture of the cuticle, along a predetermined line of weakness, visible on the apical cuticular layer (Figure 4e and Figure 5e–f). The cuticular envelope appeared broken to several degrees in many of the mature glands. Fractured cuticles also revealed the flocculent appearance of the secreted materials within the subcuticular chamber (Figure 4e).

No previous micromorphological contribution was found on the target species, while the literature data referred to works focused on European and Western Asian congeneric species. Secretory canals and both covering and glandular trichomes were described on both vegetative and reproductive organs by light and electron microscopy [16,17,18,19,20,21]. The canals were never thoroughly investigated and the literature reported only generic references [16,18], whereas information regarding glandular trichomes was more detailed. We did not detect secretory canals in the examined samples, while the biseriate, 10-celled glandular trichomes that resulted were consistent with those already known for *Achillea* genus [16,17,18,19,20,21]. Finally, the morphological features of the covering hairs, both the short digitiform and the long filamentous, were invariably uniform to the literature data, regardless of their localization on the different plant parts [16,17,18,19,21].

#### 2.3.3. Histochemistry

**Figure 8:**
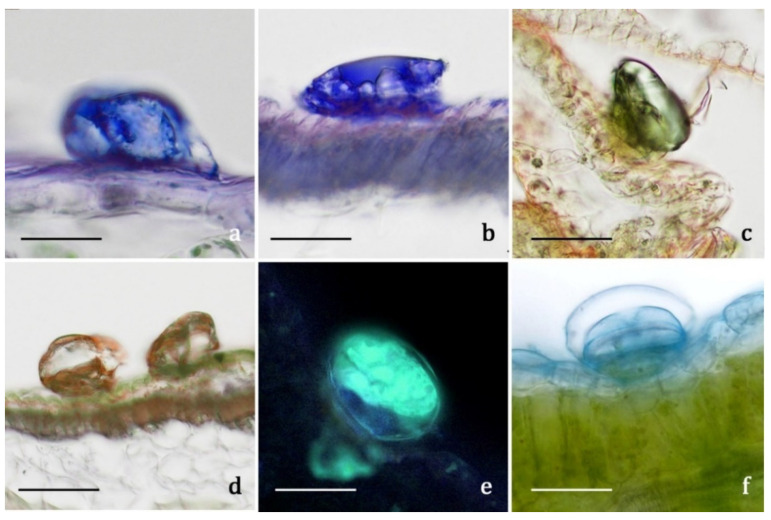
LM and FM micrographs showing the histochemical results on the secretory materials of the biseriate glandular trichomes of *Achillea moschata*. (**a**,**b**) Nadi reagent. (**c**,**d**) Ferric Trichloride test. (**e**) Aluminum Trichloride test. (**f**) Alcian Blue test. Scale bars: 30 μm (**a**,**b**,**e**,**f**); 50 μm (**c**,**d**).

The secretion products of the biseriate glandular trichomes exhibited positive responses both to lipophilic tests, such as Fluoral Yellow-088 and Nadi reagent (Figure 8a,b), and to the FeCl_3_ test (Figure 8c,d), specific to polyphenols, and to the AlCl_3_ test (Figure 8e) and Naturstoff reagent A specific to flavonoids. They showed weak coloration or negative responses to the hydrophilic dyes Alcian Blue (Figure 8f) and Ruthenium Red. Concerning internal tissues, the histochemical observations turned out to be uneven, since the results were generally negative for all the employed dyes, with sporadic weak positive responses only for polyphenols. Only one previous histochemical investigation was performed on a congeneric species, *A. millefolium*, and the production of monoterpenes was ascertained [17]. The secretory products are released externally after cuticle rupture: the volatile terpenoids emitted in the air are responsible for the typical aroma of *A. moschata*, while the polyphenolic/flavonoidic components presumably cover the epidermis constituting epicuticular exudates. Considering the overall micromorphological results, we exclude the presence of internal secretory structures, as well as the massive accumulation at tissue level of the polyphenols and flavonoids detected in the aqueous and methanolic extracts. The main sites of synthesis, accumulation, and release of such substances, along with volatile terpenoids, were represented by the biseriate glandular trichomes, distributed on the whole plant epidermal surfaces, being particularly abundant on the inflorescences (involucral bracts, interfloral scales, corollas, and ovaries of both disk and ray florets).

These observations were consistent with previous contributions on congeneric species. Some authors have evaluated the variability regarding the density of glandular trichomes on different organs (flowering stems, leaves, and florets) at different stages of development (pre-anthesis and anthesis). It was documented that the overall density increased with the advancing of the phenological stages, from the pre-anthesis phase to complete anthesis. Furthermore, the highest density rates were found for florets, followed by leaves and stems [16,18]. Therefore, the studies on trichome density confirmed that the local population collects the drug at the exact balsamic time.

In addition, based on these results, it is reasonable to hypothesize that the compounds characterizing the aqueous extract are stored at the level of these epidermal appendages.

### 2.4. Biological Activity

**Figure 9:**
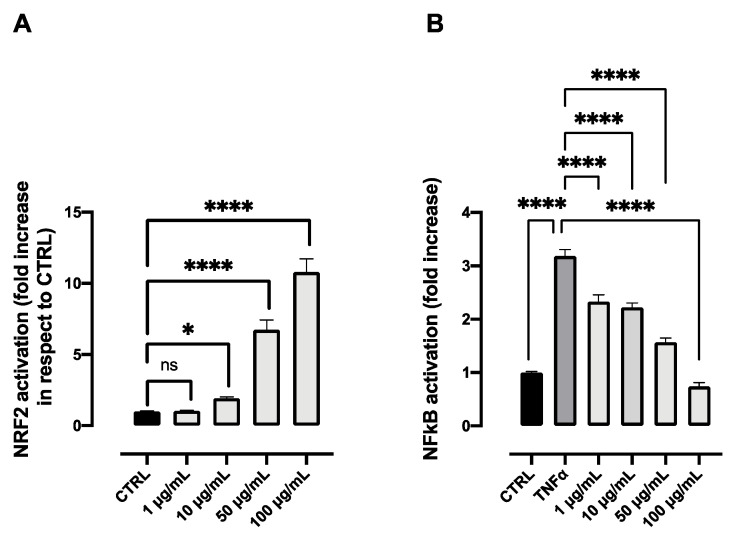
NRF2 activation (**A**) and anti-inflammatory activity (**B**) of different concentrations of the aqueous extract obtained from the flower head of *Achillea moschata*. Data are reported as mean ± SD. The statistical significance difference of each concentration in respect to the control was analyzed by one-way ANOVA followed by the Bonferroni post-test. * *p* < 0.05, **** *p* < 0.0001, ns = not significant.

The aqueous extract was evaluated for its antioxidant and anti-inflammatory potential in two different cell lines. Regarding the antioxidant activity, we tested the ability of the extract to activate the response of NRF2, a transcription factor associated with antioxidant enzymes, and which plays a master role in redox homeostasis in the cells. Data reported in Figure 9A show significant increase of NRF2 activation compared to the control. The activity is already significant at 10 μg/mL, while at the highest tested dose, 100 μg/mL, the increment is even higher than the one registered with BDX (Bardoxolone), a synthetic NRF2 activator used as a positive control. Concerning the anti-inflammatory activity, Figure 9B highlights some remarkable results. Indeed, the extract was able to significantly decrease the inflammatory response due to the stimulation of TNFα already at 1 μg/mL, while at 100 μg/mL, the response was comparable to control values. Cell viability was assessed and found to be comparable to the control values in both cell lines for all the tested concentrations (data not shown).

Notably, it was the first time that these biological activities were reported in vitro for the aqueous extract of *A. moschata*. The antioxidant activity of *A. moschata* was previously reported using the DPPH assay, demonstrating, in in vitro conditions, a direct radical scavenging activity of the polyphenolic constituents of the species [9]. However, it is now well established that in in vivo conditions, the scavenging of radicals by polyphenols, and in general by antioxidants, cannot occur due to kinetic constraints (with the exception of vitamin E as a scavenger of lipid peroxyl radicals) [32]. Hence, the in vivo antioxidant defense of small antioxidants, such as polyphenols, is not due to a direct radical scavenging mechanism but rather to the activation of the NRF2 (NF-E2-related factor 2) signaling pathway which upregulates the enzymatic removal of the precursors of radical species, such as hydroperoxides, in two-electron redox reactions. We demonstrated herein the ability of polyphenols from *A. moschata* to activate the NRF2 pathway, a mechanism which could result in an in vivo antioxidant defense mechanism.

For the anti-inflammatory activity, an in vitro study was conducted on the aqueous extract by Vitalini et al. [9] on CaCo-2 cells focusing on the modulation of the release of interleukins. However, the novelty of our work regarding the study of the anti-inflammatory mechanism, which once activated, led to an anti-inflammatory response of which the release of inflammatory interleukins is one of the effects. The activation of NFKB is only partially related to the transcription of interleukins; it is responsible for numerous other mediators [33]. A strict crosstalk between inflammation and oxidative stress and between the two transcriptional factors involved, NFKB and NRF2, is now well established. When inflammation occurs as a primary disorder, it induces oxidative stress as a secondary disorder, which can further enhance inflammation. On the other hand, when oxidative stress is induced, inflammation develops as a secondary disorder and further enhances oxidative stress [34,35]. Based on this mechanism, the anti-inflammatory activity of the aqueous extract could be related not only to its action on the NFKB pathway, but also to the activation of NRF2. Because of the striking importance these two pathways have in the regulation of anti-inflammatory and antioxidant processes, it is notable that the aqueous extract is able to significantly work even at low concentrations on both processes.

Regarding the previous studies on the potential biological activity of *A. moschata* extracts [9,11,12,15], the phenolic profile was generally investigated concerning antioxidant and anti-bacterial properties. Once again, the tested activities rarely considered the traditional uses, or they did not describe them properly [9,15]. Moreover, even in such cases as these, the tested extracts deviated from the traditional remedies documented in the considered ethnobotanical studies.

With reference to the composition of the hot aqueous extract, which faithfully reproduces the decoction traditionally used and which was firstly characterized in this work, we proposed hereafter some considerations on the available literature data. Regarding the biological activity, we performed a survey with special focus on the gastrointestinal tract. The scientific literature documented an interesting in vitro anti-inflammatory ability ascribable to some of the main compounds found in the aqueous and methanolic extracts. As for the mechanisms, a few studies conducted on apigenin 7-glucoside (15) and chlorogenic acid (2) recorded important antioxidant and radical scavenging activities. The inhibition of the expression and translocation of Nuclear factor-kappa B (NF-κB) and of the production of pro-inflammatory cytokines, such as Tumor necrosis factor-α (TNF-α) and Interleukin-8 (IL-8), was also detected [36,37,38,39,40,41]. Apigenin 7-glucoside was also considered as a potential candidate against gastric cancer progression [42], due to the positive modulation of intracellular Reactive Oxygen Species (ROS) production. Moreover, some authors attributed to chlorogenic acid the healing properties of *Taraxacum* spp. on gastrointestinal disorders such as dyspepsia and gastritis [43]. The inhibition of the nuclear translocation of NF-κB, with the subsequent dwindling of COX-2 gene expression and of Prostaglandin E2 (PGE2) production, could be the mechanism underlying the anti-inflammatory activity of dicaffeoylquinic acids (13). In addition, another mechanism can be considered, namely, the inhibition of the expression of Inducible Nitric Oxide Synthase (iNOS) and the production of Nitric Oxide (NO) [44,45]. Karbab et al. [46] ascribed to isorhamnetin 3-O-beta-glucoside, which is one of the potential compounds for peak number 16, anti-inflammatory properties related to the inhibition of protein denaturation. Little has been reported on compound number 22, apigenin 7-malonyl-glucoside, which characterizes the aqueous extract. Han et al. [47] showed that it is one of the potential NF-κB inhibitor activity components of *Flos Chrysanthemi*, while Martínez-Vázquez et al. [48] described a sedative effect of the *Dracocephalum moldavica* L. aqueous extract with a high content of apigenin- and luteolin 7-malonyl-glucosides. Malonyl-glucosides can either be hydrolyzed in the gastrointestinal tract up to their relative glucosides [49], or absorbed in their intact form [50], thus exerting their activity in both conditions.

## 3. Materials and Methods

### 3.1. Ethnobotanical Research—Interview Management and Data Archiving

From 2019 to 2021, the ethnobotanical fieldwork was conducted as reported in Bottoni et al. [1]. Open and semi-structured interviews were conducted through a questionnaire, consisting of an “Informant Sheet” and a “Species Sheet” (the summary of the questionnaire is reported in Table S3). The former was used to document the personal data of the informant and feedback of the interview, while the latter consisted of a seven-column table arranged to fix common and vernacular name of the cited species, field of use, detailed use, preparation form, administration form, part of the plant, and other information. Each “Informant Sheet” was matched to the corresponding “Species Sheet” through an alphanumeric identification code, and subsequently filed in chronological order. For each species, information was organized according to the field of use and, eventually, in more detailed categories. For example, the therapeutic field was divided into several categories, with one for every anatomical apparatus (digestive tract disorders, musculoskeletal system disorders and traumas, etc.). All data obtained from the interviews were filed in a database in the form of an Excel spreadsheet (Microsoft, Redmond, WA, USA) where each row represented a citation, defined as a single use reported for a single species by a single informant. The overall data referring to the therapeutic use of the plants for the treatment of human diseases were processed using pivot tables, basing the analysis mainly on the number of citations.

### 3.2. Plant Material

For the phytochemical and micromorfological investigations, flower heads of *Achillea moschata* were gathered in July 2019 in Alpe Mastabbia, at 2200 m a.s.l. (Chiesa in Valmalenco, Sondrio, Lombardy, Northern Italy). The collection site is characterized by a siliceous substrate and by niches with accumulations of raw humus, at windy and exposed ridges. Voucher specimens were labelled with the codes GBG118/GBG119 and deposited in the Herbarium of the Ghirardi Botanical Garden of the University of Milan (DISFARM, Toscolano Maderno, Brescia, Italy). Prof. G. Fico and Prof. C. Giuliani identified the species according to Pignatti et al. [30].

### 3.3. Phytochemical Investigation

#### 3.3.1. Preparation of the Decoction

The decoction was reproduced as documented during the ethnobotanical fieldwork, following the dosages and indications provided by the informants. To prepare the volume of a cup of tea (150–200 mL), 5–6 small bundles of 10–12 flower heads were each placed in a small pot with water. Once it boiled, it was turned off, left to rest for 3–4 min, and then filtered. Traditionally, at this point, the product was taken orally when lukewarm, with or without the addition of sugar, due to its strong bitter taste. For the laboratory research path, the decoction, once filtered, was freeze-dried. Vials (Schott, G) were filled with 10 mL of the product, semi-stoppered, and placed on the shelves of an Alpha 1–4 LSC plus freeze dryer (Martin Christ, G). Product temperature was monitored by a single wire thin thermocouple. First, samples were frozen at the rate of 0.5 K min^−1^ to a minimum shelf temperature of −20 °C that was held for 6 h to assure complete freezing. In the primary drying, samples were kept at 10 °C and 0.2 mbar for 24 h. Afterwards, the shelf temperature was increased to 25 °C at the rate of 0.1 K min^−1^ to initiate the secondary drying. The desorption phase was performed over a 6 h period.

#### 3.3.2. Preparation of the Methanolic Extract

Flower heads of *Achillea moschata* were dried at room temperature, before 45 g of the sample were extracted through three sequential extractions with increasing polarity solvents: *n*-hexane, dichloromethane, and methanol. Maceration was performed at room temperature and continued until the drug was exhausted for about 1 month for each solvent used for a total of 3 months. The methanolic extract was then stored at 4 °C until its characterization.

#### 3.3.3. Chemicals

Formic acid and LC-MS grade solvents were purchased from Merck KGaA, Darmstadt, Germany. LC-grade H_2_O (18 MΩ cm) was prepared with a Milli-Q H_2_O purification system (Millipore, Bedford, MA, USA).

#### 3.3.4. LC-MS Conditions

The extracts were dissolved in water (aqueous extract) and methanol (methanolic extract) to obtain a concentration of 4 mg/mL, then diluted in H_2_O/HCOOH, 100/0.1% *v*/*v* (mobile phase A) at the final concentration of 1 mg/mL for the analysis. The chromatographic separation was performed on a reversed-phase Agilent Zorbax SB-C18 column (150 × 2.1 mm, i.d. 3.5 µm, CPS Analitica, Milan, Italy) by an UltiMate 3000 system (Dionex) with a multistep program (80 min) of mobile phase A (H_2_O/HCOOH, 100/0.1, % *v*/*v*) and B (CH_3_CN/HCOOH, 100/0.1, % *v*/*v*) after the injection of 20 µL of each sample. An LTQ Orbitrap XL mass spectrometer (Thermo Fisher Scientific, San Jose, CA, USA) was used as a detector working in negative and positive ion mode, as described by Baron et al. [51]. Xcalibur 4.0 (Thermo Fisher Scientific, Milan, Italy) and Chromeleon Xpress 6.80 (Thermo Fisher Scientific, Milan, Italy) were used for instrument control and spectra analysis. A targeted data analysis was undertaken on a database built searching the literature for known *Achillea* compounds [9,10,11,12,14,15,29,37]: the identification was conducted by using the accurate mass and the isotopic and fragmentation patterns.

### 3.4. Morphological Investigation

#### 3.4.1. Botanical Line Drawings

Original botanical line drawings depicting the whole plant, along with details of the vegetative and reproductive organs, were realized. Special attention was paid to diacritic features described in Pignatti et al. [30]. Both macrographs of living specimens at the study area and properly prepared *exsiccata* were used for the realization of the botanical illustrations. They were drawn in continuous graphite tone and then retraced with a felt-tip fine-point pen.

#### 3.4.2. Microscopy

The micromorphological investigation under Light Microscopy (LM), Fluorescence Microcopy (FM), and Scanning Electron Microscopy (SEM) was performed on flowering stems, leaves, and flower heads collected from at least two different individuals at each of the three collection sites, concurrently with the sampling for the phytochemical investigation. At least five replicates for each of the investigated plant parts were examined to evaluate the variability in the morpho-anatomical features, as well as the type, distribution, and histochemistry of the secretory structures.

##### Light Microscopy (LM) and Fluorescence Microscopy (FM)

The micromorphological investigation under LM and FM was performed on both fresh material and fixed samples included in historesin (Technovit^®^ 7100). For the fresh material, sections of thickness ranging from 30 to 50 µm were obtained using a vibratome or a cryostat. Samples were also fixed in F.A.A. solution (Formaldehyde:Acetic Acid:Ethanol 70% = 5:5:90) for 10 days at 4 °C. Subsequently, fixed samples were washed in 70% ethanol for 12 h and then dehydrated progressively in ascending ethanol series, up to absolute. Pre-inclusion was then performed first with ethanol and historesin in 1:1 ratio for one night, then with a 1:2 ratio for 2 h, and in pure historesin for 3 h. Finally, the inclusion was performed in a polypropylene capsule with the addition of a hardener with a ratio of 1:15 of basic resin [52]. The historesin samples were cut in 2 µm sections by means of an ultramicrotome. The following dyes were employed [52]: Fluoral Yellow-88 for total lipids; Nile Red for neutral lipids; Nadi reagent for terpenes; Alcian Blue for mucopolysaccharides; Ruthenium Red for pectins; Ferric Trichloride for polyphenols; Aluminum Trichloride and Naturstoff reagent A for flavonoids. Observations were made with a Leitz DM-RB Fluo (Oberkochen, Germany) optical microscope equipped with a Nikon digital camera.

##### Scanning Electron Microscopy (SEM)

For SEM observations, small segments of the examined samples were F.A.A.-fixed for 7 days, dehydrated with ascending ethanol series up to absolute, critical-point dried, mounted on aluminum stubs, and carbon gold-coated [53]. Observations were performed under a Zeiss^®^ EVO MA15 SEM (Oberkochen, Germany) operating at 10 kV at the Interdepartmental Center for Electron Microscopy and Microanalysis Services (M.E.M.A.) of the University of Florence (Florence, Italy).

### 3.5. Biological Activity

#### 3.5.1. Antioxidant Activity

The aqueous extract was evaluated for its ability to modulate the antioxidant response pathway by monitoring the luciferase activity, strictly correlated with NRF2 activation as reported by Ferrario et al. [54] with minor changes. Experiments were performed using NRF2/ARE Responsive Luciferase Reporter HEK293 stable cell line (Signosis, Santa Clara, CA, USA) in Dulbecco’s modified Eagle medium (DMEM; Lonza, Verviers, Belgium) supplemented with 10% fetal bovine serum (FBS; Gibco, Gaithersburg, MD, USA), 1% Penicillin/Streptomycin (Lonza), and 50 µg/mL of G418 sulfate solution (Promega Corporation, Madison, WI, USA). HEK293 cells were treated with different concentrations (1, 10, 50, and 100 µg/mL) of extract for 18 h after seeding in a white 96-well plate (BRANDplates^®^, cell grade) at 10,000 cells/well. Subsequently, to avoid any interference on the reading of luciferase activity, media were removed and 50 µL/well of PBS was added. ONE-Glo™ Luciferase Assay Substrate (purchased from Promega Corporation, Madison, WI, USA) (50 µL/well) was directly added to the wells, followed by a luciferase measurement performed using a luminometer (Wallac Victor2 1420, Perkin-Elmer™ Life Science, Monza, Italy). Experiments were performed with biological and technical replicates, with results shown as mean ± SD compared to untreated control cells. Statistical analysis was performed using one-way ANOVA with Bonferroni’s multiple comparisons test (*p* < 0.05 was considered significant). The cell viability was assessed with MTT assay on HEK293 cells treated with all the concentrations of the extract tested for the antioxidant activity.

#### 3.5.2. Anti-Inflammatory Activity

The in vitro anti-inflammatory activity of the aqueous extract was evaluated using a cell model that has been previously described [55]. Briefly, R3/1-Nf-κB cells were seeded (5000 cells/well) in a white 96-well plate (BRANDplates^®^, cell grade). Cells were pretreated with different concentrations of the extracts (1, 10, 50, and 100 µg/mL) for 18 h in complete medium (DMEM 10% FBS, 1% L-glutamine, 1% Penicillin/Streptomycin). This process was followed by a 6 h stimulation with 10 ng/mL TNFα. To avoid components’ interference with the reading of the luciferase assay, cells were washed once with 100 µL of warm PBS before 50 µL of DMEM was added. Subsequently, 50 µL ONE-Glo™ Luciferase Assay Substrate (purchased from Promega Corporation, Madison, WI, USA) was directly added to the wells, followed by a luciferase measurement performed using a luminometer (Wallac Victor2 1420, Perkin-Elmer™ Life Science, Monza, Italy). Experiments were performed with biological and technical replicates and the results are shown as mean ± SD compared to untreated control cells. Statistical analysis was performed using one-way ANOVA with Bonferroni’s multiple comparisons test (*p* < 0.05 was considered significant). The cell viability for all the concentrations tested in the anti-inflammatory assay was verified by MTT assay on R3/1-Nf-κB cells.

#### 3.5.3. Cell Viability

The cell viability for all the concentrations of the aqueous extract tested was verified by MTT assay on HEK293 and R3/1-Nf-κB cells in transparent 96-well plates seeded with 10,000 and 5000 cells/well, respectively, in Dulbecco’s modified Eagle medium (DMEM; Lonza, Verviers, Belgium) supplemented with 10% fetal bovine serum (FBS; Gibco, Gaithersburg, MD, USA) and 1% Penicillin/Streptomycin (Lonza). After 18 h incubation with the extract at the appropriate concentrations (1, 10, 50, and 100 µg/mL), media were removed and, for the R3/1-NF-κB cell line, one wash with 100 µL PBS occurred. Subsequently, 50 µL/well of DMEM was added, followed by 11 µL/well MTT (3-(4,5-Dimethyl-2-thiazolyl)-2,5-diphenyl-2H-tetrazolium bromide, purchased from Merck KGaA, Darmstadt, Germany) reagent (5 mg/mL). After 4 h incubation, DMEM was removed, cells were treated with lysis buffer (100 µL/well) (HCl 8 mM, 5% TWEEN20, DMSO), and the 96-well plate was shaken for 15 min in a plate shaker in the dark. Absorbance at 575 nm and 630 nm was measured using a plate reader (BioTek’s PowerWave HT, Winooski, VT, USA). Cells incubated with media were used as a control of 100% proliferation, while cells incubated with DMSO (4%) were used as a negative control.

## 4. Conclusions

The novelty of our study was the combination of complementary research approaches: from ethnobotany to phytochemistry and morphology, up to biological activity. The ethnobotanical research conducted for the first time at the study area highlighted the traditional value of the target species. The phytochemical investigation of the aqueous extract obtained following the traditional procedure revealed a richer composition than previously described in the literature. The micromorphological investigation allowed for the first time an accurate description of flowers and inflorescences that represent the herbal drug. Moreover, the applications of histochemical dyes not only confirmed the production of phenolic derivatives, but also allowed for the enrichment of the literature data concerning the sites of synthesis and release of terpenic substances. Finally, the biological activity tests represented an important validation of the traditional use of the aqueous extract, being active at very low concentrations.

With regards to future research perspectives, the importance emerged of investigating the composition of the plant exudates, with the aim of confirming the histochemical outcomes. Indeed, this evidence allowed us to hypothesize that the accumulation of the active compounds occur at the level of the epidermal appendages. Finally, these results are consistent with the traditional use of the whole dried inflorescences, directly immersed in water without being chopped or pounded.

## Figures and Tables

**Table 1:** LC-ESI-(+/−)-MS identification of the constituents of the two extracts of *Achillea moschata*. The peak numbers refer to those reported in Figure 2. * Compounds found only in the methanolic extract.

Peak Number	Name	RT (min)	[M − H]^−^ _exp_	MS/MS	[M + H]^+^ _exp_	MS/MS
1	Caffeoylquinic acid isomer 1	3.1	353.08667	179-191	355.10245	163
2	Caffeoylquinic acid isomer 2	5.1	353.08630	161-179-191	355.10239	163
3	Vicenin-2	9.5	593.14972	353-383-473-503	595.16553	475
4	Luteolin C-glucoside *	13.7	447.09320	285-327-357	449.10782	383-353-329-287
5	Schaftoside	14.5	563.14001	353-383-443-473-503	565.15497	475-445
6	Quercetin 3-O-rutinoside (Rutin)	19.9	609.14416	301	611.16046	449-303
7	Kaempferol O-glucoside	21.2	447.09320	285	449.10779	287
8	Luteolin O-glucoside	22.1	447.09320	285	449.10782	287
9	Mearnsetin hexoside isomer 1	22.1	493.09900	316-331	495.11279	333
10	Mearnsetin hexoside isomer 2	23.3	493.09903	316-331	-	-
11	Dicaffeoylquinic acid isomer 1	26.2	515.11975	179-335-353	-	-
12	Isorhamnetin O-hexoside isomer 1	26.0	477.10420	300-315	479.11804	317
13	Dicaffeoylquinic acid isomer 2	27.6	515.11987	179-191-335-353	-	-
14	Isorhamnetin O-rutinoside	29.0	623.15900	300-315	625.17625	479-317
15	Apigenin 7-glucoside (Cosmosiin)	29.8	431.09821	269	433.11282	271
16	Isorhamnetin O-hexoside isomer 2	30.3	477.10403	300-315	479.11876	317
17	Syringetin 3-O-glucoside	33.0	507.11465	345	509.12839	347
18	Isoorientin 7-methyl ether	33.3	461.10919	284-299	463.12344	301
19	Dicaffeoylquinic acid isomer 3	33.8	515.11969	179-191-335-353	-	-
20	Luteolin 7-malonyl-glucoside	34.5	533.09357	489-285	535.10779	449-287
21	Eupatolin	36.8	491.11972	299313-314-328-329-343	493.13373	331
22	Apigenin 7-malonyl-glucoside	43.6	517.09894	473-269	519.11273	433-271
23	Luteolin	45.9	285.04028	107-133-151-175-199-217-241-257	287.05527	241-153
24	Axillarin	51.4	345.06122	271-287-300-315	347.07651	314-285-257
25	Apigenin	52.9	269.04556	107-149-225	271.06033	153-119
26	Chrysoeriol/Hispidulin	53.9	299.05542	256-284	301.0708	286-153
27	Desmethoxycentaureidin *	54.1	329.06641	299-314	331.08151	270-243
28	Isorhamnetin *	54.4	315.05066	300	317.06577	285-257-229-153-119
29	Quercetin 3,3’-dimethyl ether	54.8	329.06674	-	331.08145	301-273
30	6-Hydroxykaempferol 3,6-dimethyl ether	55.5	329.06650	-	331.08142	315-287
31	Chrysosplenol-D/Jaceidin	56.4	359.07712	314-329-341	361.09195	331

## Data Availability

Not applicable.

## Supplementary Materials

The following supporting information can be downloaded at: https://www.mdpi.com/article/10.3390/molecules27238318/s1. Table S1: Plant species traditionally used in Chiesa in Valmalenco (Sondrio, Lombardy, Northern Italy). Species are reported in decreasing order of citations. Table S2: Plant species used in the therapeutic field in Chiesa in Valmalenco (Sondrio, Lombardy, Northern Italy). Species are reported in decreasing order of citations. Table S3: Summary of the questionnaire, in both the Italian and English languages, proposed during the interviews to the local community in Chiesa in Valmalenco (Sondrio, Lombardy, Northern Italy).
