# Supplementary material for: Achillea moschata Wulfen: From Ethnobotany to Phytochemistry, Morphology, and Biological Activity
Source: Molecules. 2022 Nov 29;27(23):8318. doi: 10.3390/molecules27238318 (PMC9736511; doi:10.3390/molecules27238318)
Supplement: Supplementary file 1 [file molecules-27-08318-s001.zip › molecules-2050499-supplementary.pdf]

Supplementary Materials

Table S1. Plant species traditionally used in Chiesa in Valmalenco (Sondrio, Lombardy, Italy). Species are reported in decreasing order of citations.

N.	Species	Citations
1	*Arnica montana* L. subsp. *montana*	248
2	*Vaccinium myrtillus* L.	242
3	*Achillea moschata* Wulfen	209
4	*Taraxacum* spp.	185
5	*Sambucus nigra* L.	177
6	*Vaccinium vitis-idaea* L.	165
7	*Rubus idaeus* L.	164
8	*Thymus* spp.	151
9	*Malva sylvestris* L.	137
10	*Pinus mugo* Turra	133
11	*Urtica dioica* L.	112
12	*Rosa canina* L.	112
13	*Fragaria vesca* L.	107
14	*Artemisia genipi* Weber	101
15	*Gentiana lutea* L.	100
16	*Hypericum perforatum* L.	95
17	*Juniperus communis* L.	89
18	*Achillea millefolium* L.	83
19	*Matricaria chamomilla* L.	77
20	*Silene vulgaris* (Moench) Garcke	76
21	*Blitum bonus-henricus* (L.) Rchb.	75
22	*Calendula officinalis* L.	72
23	*Rubus ulmifolius* Schott	64
24	*Larix decidua* Mill.	63
25	*Pinus cembra* L.	56
26	*Plantago major* L.	41
27	*Rumex alpinus* L.	35
28	*Picea abies* (L.) H.Karst.	34
29	*Salvia officinalis* L.	33
30	*Aruncus dioicus* (Walter) Fernald	30
31	*Equisetum arvense* L.	23
32	*Polypodium vulgare* L.	21
33	*Plantago lanceolata* L.	20
34	*Rumex acetosa* L.	19
35	*Mentha x piperita* L.	16
36	*Carlina acaulis* L.	16

37	*Euphrasia rostkoviana* Hayne	16
38	*Ribes rubrum* L.	16
39	*Corylus avellana* L.	16
40	*Prunus spinosa* L.	16
41	*Artemisia absinthium* L.	15
42	*Humulus lupulus* L.	14
43	*Rhododendron ferrugineum* L.	14
44	*Cetraria islandica* (L.) Ach. subsp. *islandica* (“Lichen”)	14
45	*Arctium lappa* L.	12
46	*Prunus avium* L.	12
47	*Chelidonium majus* L.	12
48	*Betula pendula* Roth	12
49	*Solanum tuberosum* L.	11
50	*Brassica oleracea* L.	11
51	*Melissa officinalis* L.	11
52	*Castanea sativa* Mill.	10
53	*Fraxinus excelsior* L.	8
54	*Ribes nigrum* L.	8
55	*Juglans regia* L.	8
56	*Lavandula angustifolia* Mill.	8
57	*Allium sativum* L.	8
58	*Bistorta officinalis* Delarbre	7
59	*Tilia* sp. pl.	7
60	*Rosmarinus officinalis* L.	7
61	*Achillea nana* L.	6
62	*Crataegus monogyna* Jacq.	6
63	*Nasturtium officinale* R. Br.	6
64	*Secale cereale* L.	6
65	*Zea mays* L.	6
66	*Linum usitatissimum* L.	5
67	*Lamium album* L.	5
68	*Nigritella rhellicani* Teppner et E. Klein	5
69	*Carum carvi* L. subsp. *carvi*	5
70	*Sorbus aucuparia* L.	5
71	*Rheum officinale* L.	5
72	*Alnus viridis* (Chaix) DC.	5
73	*Polygonum aviculare* L.	4
74	*Triticum aestivum* L.	4
75	*Trifolium pratense* L.	4
76	*Gentiana acaulis* L.	4

77	*Hordeum vulgare* L.	4
78	*Chaerophyllum aromaticum* L.	4
79	*Laurus nobilis* L.	4
80	*Allium schoenoprasum* L.	3
81	*Gentiana verna* L.	3
82	*Capsicum annuum* L.	3
83	*Robinia pseudoacacia* L.	3
84	*Ocimum basilicum* L.	3
85	*Sambucus racemosa* L.	3
86	*Artemisia vulgaris* L.	3
87	*Calluna vulgaris* (L.) Hull	3
88	*Carduus nutans* L.	3
89	*Cinnamomum verum* J. Presl	3
90	*Aloe vera* (L.) Burm. fil.	3
91	*Solanum lycopersicum* L.	3
92	*Olea europaea* L.	3
93	*Pinus sylvestris* L.	3
94	*Prunus domestica* L.	3
95	*Oxalis acetosella* L.	3
96	*Malus x domestica* Borkh.	3
97	*Leontopodium alpinum* Cass.	3
98	*Berberis vulgaris* L.	2
99	*Sedum album* L.	2
100	*Elymus repens* L. Gould	2
101	*Heracleum sphondylium* L.	2
102	*Panicum miliaceum* L.	2
103	*Brassica rapa* L.	2
104	*Salix alba* L.	2
105	*Allium cepa* L.	2
106	*Fagus sylvatica* L.	2
107	*Capsella bursa-pastoris* (L.) Medik.	2
108	*Ficus carica* L.	2
109	*Ricinus communis* L.	2
110	*Phyteuma betonicifolium* Vill.	2
111	*Lepidium sativum* L.	2
112	*Verbascum thapsus* L.	2
113	*Epilobium montanum* L.	2
114	*Matricaria discoidea* DC.	2
115	*Gentiana* spp.	2
116	*Viola odorata* L.	1

117	*Galium odoratum* (L.) Scop.	1
118	*Carlina* spp.	1
119	*Potentilla indica* (Andrews) Th. Wolf	1
120	*Pinus* sp. pl.	1
121	*Lupinus albus* L.	1
122	*Oryza sativa* L.	1
123	*Arctostaphylos uva-ursi* (L.) Spreng.	1
124	*Physalis alkekengi* L.	1
125	*Aesculus hippocastanum* L.	1
126	*Ajuga pyramidalis* L.	1
127	*Cirsium* spp.	1
128	*Abies alba* Mill.	1
129	*Pteridium aquilinum* (L.) Kuhn subsp. *aquilinum*	1
130	*Prunus cerasus* L.	1
131	*Tagetes* spp.	1
132	*Daphne mezereum* L. subsp *mezereum*	1
133	*Alchemilla pentaphyllea* L.	1
134	*Cichorium intybus* L.	1
135	*Alliaria petiolata* (M.Bieb.) Cavara et Grande	1
136	*Tropaeolum majus* L.	1
137	*Sempervivum tectorum* L.	1
138	*Diplotaxis tenuifolia* (L.) DC.	1
139	*Cucurbita maxima* Duchesne	1
140	*Veratrum album* L.	1
141	*Nicotiana tabacum* L.	1
142	*Verbena officinalis* L.	1
143	*Foeniculum vulgare* Mill.	1
144	*Vitis vinifera* L.	1
145	*Sorbus aria* (L.) Crantz	1
146	*Allium ursinum* L.	1
147	*Fagopyrum esculentum* Moench	1
148	*Sedum album* L.	1
	**Total**	**3870**

Table S2. Plant species used in the therapeutic field in Chiesa in Valmalenco (Sondrio, Lombardy, Italy). Species are reported in decreasing order of citations.

N.	Species	Citations
1	*Arnica montana* L. subsp. *montana*	248
2	*Achillea moschata* Wulfen	132
3	*Malva sylvestris* L	115
4	*Pinus mugo* Turra	96
5	*Hypericum perforatum* L.	93
6	*Achillea millefolium* L.	70
7	*Matricaria chamomilla* L.	70
8	*Sambucus nigra* L.	64
9	*Thymus* spp.	62
10	*Calendula officinalis* L.	62
11	*Taraxacum* spp.	34
12	*Gentiana lutea* L.	31
13	*Plantago major* L.	26
14	*Rosa canina* L.	25
15	*Equisetum arvense* L.	23
16	*Juniperus communis* L.	22
17	*Vaccinium myrtillus* L.	21
18	*Vaccinium vitis-idaea* L.	20
19	*Rumex alpinus* L.	18
20	*Salvia officinalis* L.	16
21	*Picea abies* (L.) H.Karst.	16
22	*Euphrasia rostkoviana* Hayne	15
23	*Urtica dioica* L.	14
24	*Cetraria islandica* (L.) Ach. subsp. *islandica* ("Lichen")	14
25	*Artemisia genipi* Weber	13
26	*Larix decidua* Mill.	13
27	*Chelidonium majus* L.	12
28	*Pinus cembra* L.	11
29	*Arctium lappa* L.	9
30	*Plantago lanceolata* L.	8
31	*Brassica oleracea* L.	8
32	*Melissa officinalis* L.	8
33	*Solanum tuberosum* L.	7
34	*Allium sativum* L.	6
35	*Artemisia absinthium* L.	6
36	*Linum usitatissimum* L.	5
37	*Chaerophyllum aromaticum* L.	4

38	*Rheum officinale* Baill.	4
39	*Triticum aestivum* L.	3
40	*Capsicum annuum* L.	3
41	*Tilia* sp. pl.	3
42	*Cinnamomum verum* J. Presl	3
43	*Hordeum vulgare* L.	3
44	*Olea europaea* L.	3
45	*Gentiana verna* L.	3
46	*Ricinus communis* L.	2
47	*Ribes nigrum* L.	2
48	*Fraxinus excelsior* L.	2
49	*Pinus sylvestris* L.	2
50	*Panicum miliaceum* L.	2
51	*Prunus avium* L.	2
52	*Secale cereale* L.	2
53	*Verbascum thapsus* L.	2
54	*Elymus repens* L. Gould	2
55	*Capsella bursa-pastoris* (L.) Medik.	2
56	*Mentha x piperita* L.	2
57	*Veratrum album* L.	1
58	*Nicotiana tabacum* L.	1
59	*Arctostaphylos uva-ursi* (L.) Spreng.	1
60	*Epilobium montanum* L.	1
61	*Oryza sativa* L.	1
62	*Pinus* sp. pl.	1
63	*Verbena officinalis* L.	1
64	*Sempervivum tectorum* L.	1
65	*Nasturtium officinale* R. Br.	1
66	*Betula pendula* Roth	1
67	*Aloe vera* (L.) Burm.fil.	1
68	*Sorbus aucuparia* L.	1
69	*Aruncus dioicus* (Walter) Fernald	1
70	*Gentiana* spp.	1
71	*Tagetes* spp.	1
72	*Lupinus albus* L.	1
73	*Humulus lupulus* L.	1
74	*Sedum album* L.	1
	**Total**	**1482**

Table S3. Summary of the questionnaire, in Italian and in English languages, proposed during the interviews to the local community in Chiesa in Valmalenco (Sondrio, Lombardy, Italy).

QUESTIONARIO

(intervista aperta o semi-strutturata)

A) INFORMAZIONI GENERALI

1. Chi intervista
2. Data e luogo intervista
3. Intermediario
4. Intervistato
5. Età
6. Grado di parentela
7. Professione
8. Recapito dell'informatore o dell'intermediario

B) INFORMAZIONI SULLE PIANTE E LORO USI

1. Usi o conosci qualche pianta?
2. Nome comune della pianta (*anche dialettale*)
3. Come la usi? Per cucinare o per curarti?
4. Per quale disturbo la usi? (*medicinale*)
5. Quale parte della pianta usi?
6. Come la prepari?
7. Come la somministri? (*medicinale*)
8. Dove e quando la raccogli?
9. Da chi hai imparato a farne uso?
10. La usi personalmente o ne hai solo sentito parlare?
11. È un uso ancora attuale o passato?

QUESTIONNAIRE

(open and semi-structured interview)

C) GENERAL INFORMATION

9. Interviewer's name and surname

10. Date and place of the interview

11. Mediator

12. Informant's name and surname

13. Age

14. Degree of kinship

15. Job (current or past)

16. Mediator or informant's phone number

D) INFORMATION ABOUT PLANT SPECIES AND THEIR TRADITIONAL USES

12. Do you use or know any plant species?

13. Common name of the plant species (*even its dialectal name*)

14. How do you use it? Is it useful for cooking or for medicinal purposes?

15. What ailment do you use it for? (*therapeutic field of use*)

16. Which plant's part do you use?

17. How do you prepare it?

18. How do you administer it? (*therapeutic field of use*)

19. Where and when do you collect it?

20. Who did you learn to use from?

21. Do you use it personally or have you just heard of it?

22. Is it still a current or past use?
